# Estimulação Cardíaca Parahissiana – Nova Alternativa de Estimulação mais Fisiológica do Coração?

**DOI:** 10.36660/abc.20220016

**Published:** 2022-02-14

**Authors:** Rodrigo M. Kulchetscki, Mauricio Scanavacca

**Affiliations:** InCor HC FMUSP São Paulo SP Brasil Unidade Clínica de Arritmias – InCor - HC - FMUSP , São Paulo , SP – Brasil

**Keywords:** Estimulação Cardíaca Artificial/instrumentação, Marca-Passo Artificial/tendências, Terapia por Estimulação Elétrica/tendências

Desde o implante do primeiro marca-passo cardíaco em 1958 ^1^ a estimulação cardíaca artificial tem sido alvo de inumeráveis pesquisas, grande aprimoramento técnico e inovações tecnológicas. Assim, a correção de bradiarritmias por meio de dispositivos eletrônicos implantáveis é a área na qual se observou uma das maiores evoluções do conhecimento dentro da Cardiologia Intervencionista.

Mais recentemente, a descrição de uma nova técnica, a chamada estimulação cardíaca fisiológica, causou grande entusiasmo aos especialistas e uma verdadeira transformação na evolução dos pacientes. A estimulação fisiológica inclui um conjunto de métodos destinados a estimular eletricamente, direta ou indiretamente, o sistema de condução intraventricular do coração. O seu grande benefício é minimizar os efeitos deletérios causados pela estimulação direta do miocárdio ventricular direito (VD), que gera dissincronia e possível disfunção contrátil do ventrículo esquerdo em médio e longo prazo. ^2 , 3^

Scherlag et al. ^4^ descreveram as primeiras estratégias de estimulação do Feixe de His em 1967. Porém, a sua implementação na prática clínica, infelizmente só ocorreu no início dos anos 2000, por Deshmukh e colaboradores. ^5^

O sucesso das técnicas de implante (80-95%), assim como os resultados observados, vem ampliando a indicação desse modo de estimulação em diversas condições clínicas. ^6^ Sua utilização possibilitou a redução no desenvolvimento de disfunção ventricular e menor incidência de fibrilação atrial, segundo dados da literatura, ^7^ tanto na estimulação seletiva quanto na não seletiva do Feixe de His. ^8^

As limitações do método, por enquanto, incluem um tempo maior de fluoroscopia e as taxas de sucesso ainda são variáveis, dependendo da curva de aprendizado dos profissionais habilitados. Ainda, observa-se menor sensibilidade de onda R e maior incidência de aumento de limiar e perda de captura ao longo do seguimento clínico, o que justificou o implante de um eletrodo adicional no ventrículo direito por alguns profissionais.

A estimulação mais seletiva do ramo esquerdo, proposta em 2017 por Huang et al., ^9^ vem sendo utilizada num número crescente de pacientes como alternativa à estimulação hissiana, pois evita algumas dificuldades técnicas descritas no implante do eletrodo no feixe de His. ^9^

Os mesmos autores publicaram uma série envolvendo um número expressivo de pacientes, nos quais obtiveram sucesso no implante em 97,8% dos casos. Nesse estudo, observaram manutenção dos limiares de estimulação e sensibilidade de onda R semelhantes às da estimulação cardíaca artificial convencional, com a vantagem de evitar disfunção ventricular num seguimento médio de 2 anos. ^10^

A técnica de estimulação do ramo esquerdo parece requerer uma curva de aprendizado mais rápida, porém ainda exige treinamento específico e uso de materiais especiais, como bainhas pré-moldadas ou deflectíveis, além de eletrodos especiais. Estes insumos implicam em custos adicionais que podem dificultar a sua implantação rotineira na prática clínica.

Nessa edição dos Arquivos Brasileiros de Cardiologia, Ferrari et al. ^11^ propõem um novo método de estimulação cardíaca fisiológica não seletiva parahissiana. De acordo com os autores, a técnica utiliza um eletrodo de estimulação ventricular convencional, posicionado na região septal alta do ventrículo direito, próximo à localização anatômica do Feixe de His.

Nesse estudo, a estimulação foi capaz de capturar o miocárdio do ventrículo direito e, simultaneamente o sistema de condução. Para avaliar a sincronia de ativação ventricular, os autores utilizaram um software especial para análise eletrocardiográfica da variância espacial do complexo QRS. Por meio desse método, demonstraram correção da dissincronia ventricular do paciente, quando comparada ao período pré-implante.

As vantagens do método incluem menores custos, dada sua factibilidade com utilização de eletrodos convencionais de marca-passo, uma mais rápida curva de aprendizado e, aparentemente, maior sensibilidade da onda R em comparação aos implantes no Feixe de His. Por outro lado, entre as possíveis desvantagens, pontua-se a necessidade de medida da dissincronia ventricular durante o implante, por meio de um software ainda não amplamente disponível.

Por tratar-se de um estudo de pequeno porte e com tempo de seguimento apenas intrahospitalar, é pertinente questionarmos se de fato, como é sugerido pelos autores, a sincronia cardíaca medida pelo método de análise de variância espacial do QRS é suficiente para garantir uma ativação cardíaca fisiológica, a despeito do alargamento do QRS.

Os autores, no entanto, propõem essa alternativa bastante interessante, a estimulação indireta do sistema de condução, que certamente merece ser explorada em estudos com maior número de pacientes e mais tempo de acompanhamento.
