# Supplementary material for: Estimulação Cardíaca Parahissiana – Nova Alternativa de Estimulação mais Fisiológica do Coração?
Source: Arq Bras Cardiol. 2022 Feb 14;118(2):503–4. [Article in Portuguese] doi: 10.36660/abc.20220016 (PMC8856690; doi:10.36660/abc.20220016)
Supplement: Translation

# Translation: Parahissian Cardiac Stimulation – New Alternative for More Physiological Stimulation of the Heart?

**Authors:** Rodrigo M. Kulchetscki, Mauricio Scanavacca

**Affiliations:** Brazil Unidade Clínica de Arritmias – InCor-HC-FMUSP, São Paulo, SP – Brazil

**Keywords:** Eletric Stimulation Cardiac/instrumentation, Pacemarkr, Artificial/trends, Therapy Electric Stimulation/trends

Since the implantation of the first cardiac pacemaker in 1958,^1^ artificial cardiac pacing has been the subject of innumerable research, great technical improvement, and technological innovations. Thus, the correction of bradyarrhythmias using implantable electronic devices is the area in which one of the greatest advances in knowledge within Interventional Cardiology has been observed.

More recently, the description of a new technique, the so-called physiological cardiac stimulation, caused great enthusiasm among specialists and a real transformation in the evolution of patients. Physiological stimulation includes a set of methods intended to electrically stimulate, directly or indirectly, the intraventricular conduction system of the heart. Its great benefit is to minimize the deleterious effects caused by direct stimulation of the right ventricular myocardium (RV), which generates dyssynchrony and possible left ventricular contractile dysfunction in the medium and long term.^2 , 3^

Scherlag et al.^4^ described the first strategies for stimulating the Bundle of His in 1967. However, its implementation in clinical practice, unfortunately, only took place in the early 2000s.,by Deshmukh et al.^5^

The success of implant techniques (80-95%) and the results observed have been expanding the indication of this stimulation mode in different clinical conditions.^6^ Its use enabled a reduction in the development of ventricular dysfunction and a lower incidence of atrial fibrillation, according to literature data,^7^ both in selective and non-selective stimulation of the Bundle of His.^8^

The limitations of the method, for now, include a longer fluoroscopy time, and success rates are still variable, depending on the learning curve of qualified professionals. Furthermore, lower R-wave sensitivity and higher incidence of threshold increase, and loss of capture were observed during the clinical follow-up, which justified some professionals’ implantation of an additional electrode in the right ventricle.

More selective stimulation of the left branch, proposed in 2017 by Huang et al.,^9^ has been used in an increasing number of patients as an alternative to Hissian stimulation, as it avoids some technical difficulties described in the implantation of the electrode in the Bundle of His.^9^

The same authors published a series involving a significant number of patients in which the implant was successful in 97.8% of the cases. In this study, they observed maintenance of stimulation thresholds and R-wave sensitivity similar to conventional artificial cardiac pacing, with the advantage of avoiding ventricular dysfunction in a mean follow-up of 2 years.^10^

The left branch stimulation technique seems to require a faster learning curve. However, it still requires specific training and the use of special materials, such as pre-shaped or deflectable sheaths, in addition to special electrodes. These inputs imply additional costs that can make their routine implementation in clinical practice difficult.

In this edition of the *Arquivos Brasileiros de Cardiologia* , Ferrari et al.^11^ propose a new non-selective parahissian physiological cardiac pacing method. According to the authors, the technique uses a conventional ventricular pacing electrode, positioned in the high septal region of the right ventricle, close to the anatomical location of the Bundle of His.

In this study, the stimulation was able to capture the right ventricular myocardium and, simultaneously, the conduction system. To assess the timing of ventricular activation, the authors used special software for electrocardiographic analysis of the spatial variance of the QRS complex. Through this method, they demonstrated correction of the patient’s ventricular dyssynchrony compared to the pre-implant period.

The method’s advantages include lower costs, given its feasibility with conventional pacemaker electrodes, a faster learning curve and, apparently, greater R-wave sensitivity compared to His Bundle implants. On the other hand, among the possible disadvantages is the need to measure ventricular dyssynchrony during implantation using software that is not yet widely available.

As this is a small study with only in-hospital follow-up time, it is pertinent to question whether, as suggested by the authors, cardiac synchrony measured by the QRS spatial variance analysis method is sufficient to guarantee activation physiological heart rate, despite the widening of the QRS.

The authors, however, propose this very interesting alternative, indirect stimulation of the conduction system, which certainly deserves to be explored in studies with a greater number of patients and longer follow-up.
